# Venoarterial Membrane Oxygenation in Cardiogenic Shock Complicated from an Acute Myocardial Infarction: An Overview and Comprehensive Meta-Analysis

**DOI:** 10.3390/biomedicines13010237

**Published:** 2025-01-20

**Authors:** Kiarash Sassani, Styliani Syntila, Christian Waechter, Julian Kreutz, Birgit Markus, Nikolaos Patsalis, Bernhard Schieffer, Georgios Chatzis

**Affiliations:** Department of Cardiology, Angiology and Intensive Care, Philipps University Marburg, 35043 Marburg, Germany; kiarash.sassani@gmail.com (K.S.); styliani.syntila@staff.uni-marburg.de (S.S.); christian.waechter@staff.uni-marburg.de (C.W.); patsalis@med.uni-marburg.de (N.P.);

**Keywords:** cardiogenic shock, meta-analysis, VA-ECMO, ECLS, myocardial infarction

## Abstract

**Background:** Cardiogenic shock remains a significant cause of mortality in patients with acute coronary syndrome, despite early interventions, such as coronary revascularization. Mechanical circulatory support devices, particularly venoarterial extracorporeal membrane oxygenation (VA-ECMO), are increasingly being utilized to address this issue. Limited randomized controlled trials (RCTs) exist to evaluate the efficacy of VA-ECMO in cardiogenic shock related to acute coronary syndrome. **Methods:** A meta-analysis was conducted to assess the effectiveness of VA-ECMO in adult patients with infarct-related cardiogenic shock. Trials were identified through database searches and selected based on specific inclusion criteria. The primary outcome was 30-day all-cause mortality, with secondary outcomes including bleeding and vascular complications. **Results:** A total of 24 studies met the inclusion criteria and were included in the meta-analysis, involving 4706 patients. The median age of the patients was 61.8 ± 4.1 years, with 76% of them being males. The analysis revealed that 30-day mortality rates for patients with cardiogenic shock receiving ECMO were still high, with a mortality of 63%. Vascular complications were identified as factors associated with a worse prognosis. **Conclusions:** The meta-analysis highlights the ongoing challenge of high mortality rates in cardiogenic shock patients despite the use of VA-ECMO. While VA-ECMO shows promise in providing circulatory support, further research is needed to explore ways to improve outcomes and reduce complications associated with the use of these devices. The complexity of patient management in cardiogenic shock cases underscores the need for a multidisciplinary approach to optimize treatment strategies and enhance patient outcomes.

## 1. Introduction

Mortality rates are still high in patients with cardiogenic shock (CA) related to acute coronary syndrome (ACS), even after the implementation of early invasive measures of coronary revascularization in the central role of the therapy cascade [1]. As a result, mechanical circulatory support (MCS) devices, such as venoarterial extracorporeal membrane oxygenation (VA-ECMO), are increasingly being used [2]. The only MCS that can provide complete respiratory and circulatory support currentlyis extracorporeal membrane oxygenation (ECMO), which is a form of extracorporeal life support (ECLS) that provides oxygenation, carbon dioxide (CO_2_) removal, and/or circulatory support, excluding cardiopulmonary bypass [3]. With ECMO, a drainage cannula is used to remove blood from a cardiac chamber or a large central blood vessel. A semipermeable membrane is used to pump that blood through. With the use of a reinfusion cannula, blood is directly oxygenated and CO_2_ is removed from the membrane lung and returned to a large blood vessel or cardiac chamber. Thereby, both gas exchange and circulatory support with perfusion of end organs are provided by re-infused blood that combines with the natural circulation. The ECMO blood flow rate, or the rate at which blood enters the membrane, is primarily controlled manually and usually ranges from 3 to 7 L/min. This blood flow rate determines the degree of oxygenation obtained from the circuit. Furthermore, at high blood flow rates, the sweep gas flow rate and, at lower blood flow rates, the combined sweep gas and blood flow rates determine the extent to which CO_2_ is removed by the circuit. The fraction of oxygen that is delivered to the membrane lung is known as the fraction of delivered oxygen (FDO_2_), which is delivered to the native lung via mechanical ventilation and is typically set at 100 percent [4].

There are various indications for the application of ECMO. Patients with left-sided or right-sided heart failure (such as from right ventricular infarction, massive pulmonary embolism, or pulmonary hypertension), as well as those who require assistance during cardiopulmonary resuscitation and those who have refractory shock from other causes, such as trauma, anaphylaxis, drowning, organ donation, poisoning, or hypothermia, are typically the patients who may benefit from support of VA-ECMO. The goal of VA-ECMO, under those circumstances, is circulatory support, but patients may also derive benefit from extracorporeal oxygenation and CO_2_ removal if needed [5].

With the exception of acute hypoxemic respiratory failure brought on by acute respiratory distress syndrome (ARDS), supporting data for the use of ECMO in shock situations are generally lacking and primarily derived from observational case series. Currently, there are no specific guidelines dedicated to the use of VA-ECMO in cardiogenic shock (CS). Its utilization is based on recommendations from scientific panels and is generally limited to patients with severe refractory forms of shock [6]. On the other hand, due to the small number of available randomized clinical trials (RCTs), the clinical evidence level regarding the potential benefit of applying VA-ECMO in CS related to ACS remains low. Furthermore, the current trials lacked sufficient power to detect differences in survival due to small or moderate sample sizes [7,8].

In order to address the aforementioned limitations and to assess this gap in the literature, we performed this meta-analysis of trials investigating VA-ECMO in this clinical setting of ACS-related CS. All-cause death as the main outcome was examined for the entire cohort as well as for predefined subgroups.

## 2. Materials and Methods

### 2.1. Study Selection

The trials of possible interest were found through searches on the databases MEDLINE via PubMed, Cochrane Central Register of Controlled Trials, and Embase. Therefore, the following search term groups were utilized, of which each term had to match at least once:

(1) Shock, cardiogenic, cardiac shock, or abrupt loss of heart function or acute cardiac failure [Title/Abstract];
(2) Myocardial infarction or myocardial infarct or coronary infarct [Title/Abstract];
(3) Extracorporeal membrane oxygenation or ECLS or extracorporeal life support or veno(-)arterial extracorporeal membrane oxygenation or veno(-)arterial ECMO [Title/Abstract].

The search was supplemented by a sensitivity- and precision-maximizing string from Cochrane.

Permitted for inclusion in this meta-analysis were trials using VA-ECMO in adult patients with infarct-related CS, with or without ongoing mechanical cardiopulmonary resuscitation. In the event that participants in a trial had CS, only patients with myocardial infarction in the subset or main population were included in the dataset, excluding those with other etiologies of CS. Each of the included trials had its own ethical approval, as described in them.

Following the elimination of duplicates, the search item titles and abstracts were examined and subsequently eliminated in accordance with the above eligibility requirements. When there was still uncertainty after the title and abstract screening, two investigators independently examined the full-text articles.

### 2.2. End-Points and Definitions

In the intention-to-treat population, 30-day all-cause death was the main enquired outcome. Major vascular access complications were defined as critical limb ischemia requiring device removal or vascular/surgical intervention. Major bleeding was considered as bleeding BARC > 2, TIMI Major, or GUSTO severe bleeding [9]. The quality of the included studies was independently appraised by 2 reviewers (KS and GC), with disagreements resolved by consensus. For each included paper, we evaluated the risk of bias (low, unclear, or high) for random-sequence generation, allocation concealment, blinding of patients and physicians, blinding during assessment of follow-up, incomplete outcome evaluation, and selective reporting, in keeping with the Cochrane Collaboration approach. The incidence of moderate to severe bleeding and peripheral ischemic vascular complications were the secondarily assessed outcomes.

**Figure 1:**
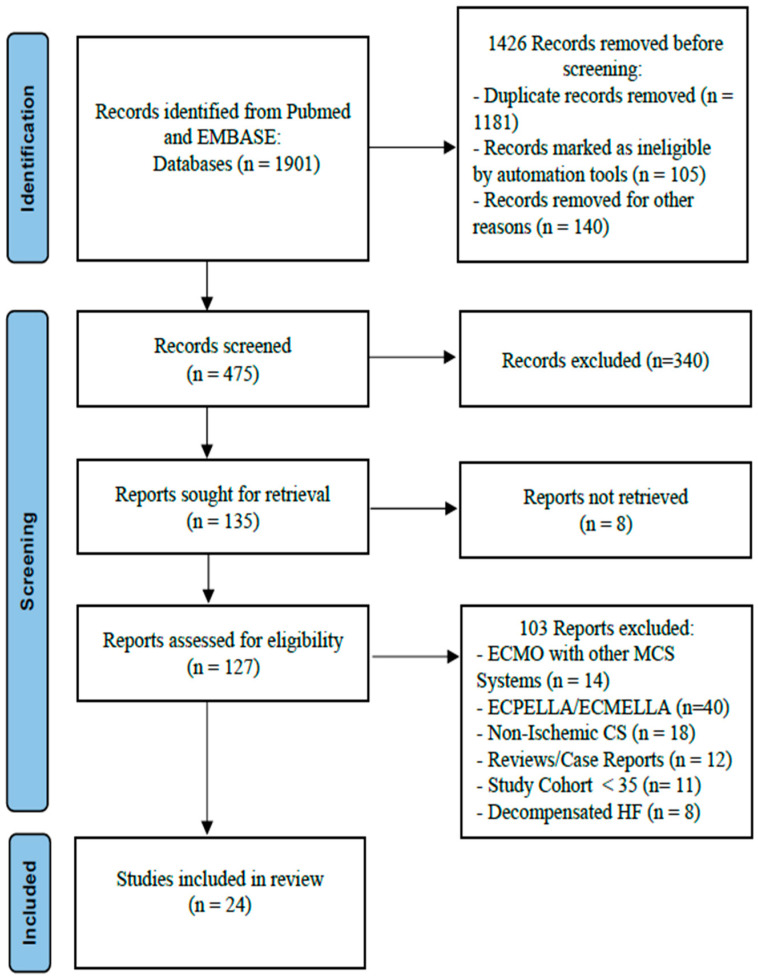
Flow chart of the studies included. ECMO: extracorporeal membrane oxygenation; CS: cardiogenic shock; HF: heart failure; MCS: mechanical circulatory support.

After searching electronic databases, 1901 items were discovered. After removing duplicates, inappropriate studies, reviews, and meta-analyses, 475 studies were left for additional evaluation. Excluded from the further qualitative analysis were reviews, meta-analyses, and trials with different etiologies of cardiogenic shock. A total of twenty-five trials have been included in this meta-analysis; studies with fewer than thirty-five cases or the population of the evaluated trials combining ECMO with other MCS-Systems were also excluded (Figure 1). Table 1 lists the number of patients from each of the 24 trials that made up the meta-analysis. It also includes the study design, publication date, and clinical endpoints for each trial.

### 2.3. Statistical Analysis

Continuous variables are reported as mean (SD) or median (first and third quartile). Categorical variables are expressed as n (%). Statistical pooling for incidence estimates was performed according to a common-effect model with generic inverse-variance weighting, computing risk estimates with 95% confidence intervals (CIs), using R Project for statistical computing (Version 4.4.1). Small study bias was assesed by graphical inspection of funnel plots. Meta-regression analysis and leave-one-out analysis were performed to assess the impact of baseline features on the primary endpoint with Comprehensive Meta-analysis software (trial version). Hypothesis testing for statistical homogeneity was set at the 2-tailed 0.10 level and based on the Cochran Q test, with I^2^ values of 25%, 50%, and 75% representing mild, moderate, and severe heterogeneity, respectively.

## 3. Results

A total of 24 retrospective observational studies (involving 4706 patients with CS and ECMO implantation) were analyzed in this systematic review (refer to Figure 1 and Table 1). The average age of participants across all studies was 61.8 ± 4.1 years, with 74.7% of patients being male. Information on prior cardiopulmonary resuscitation (CPR) was not universally reported across all studies; however, it ranged widely from 43% to 81%, indicating a cohort of patients with significant illness severity.

**Figure 2:**
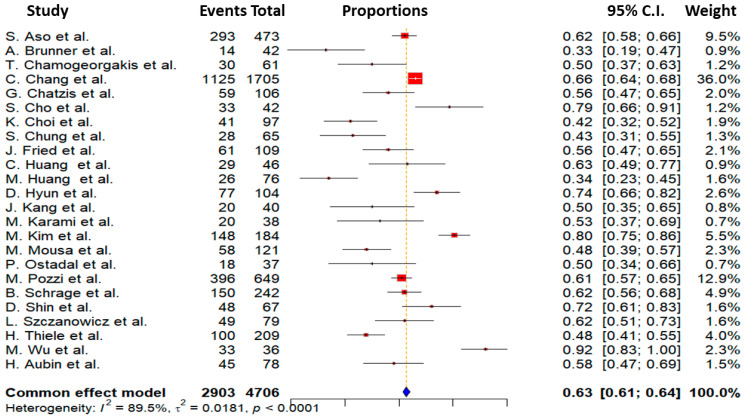
Forest plot of studies included in mortality outcomes [7,10,11,12,13,14,15,16,17,18,19,20,21,22,23,24,25,26,27,28,29,30,31,32].

Admission lactate levels were inconsistently documented in the studies but typically fell within the range of 5.6 to 14 mmol/L, suggesting a state of profound shock. Furthermore, the available data on ejection fraction as an indicator of left ventricular function on admission indicated severe impairment, with values ranging from 21% to 45%. The data on ECMO insertion in terms of coronary intervention was insufficient for a comparative analysis between pre- and post-PCI groups. An analysis of the studies included is presented in Table 2. The key finding of this analysis was a mean mortality rate of 63% among all participants, with individual rates ranging from 33% to 92%. This mortality rate was observed within 30 days of initial admission, which may account for the relatively high mortality despite the utilization of ECMO support. The study-specific mortality rates from the studies that were included included in this meta-analysis are displayed in Figure 2.

### Secondary End Points

**Figure 3:**
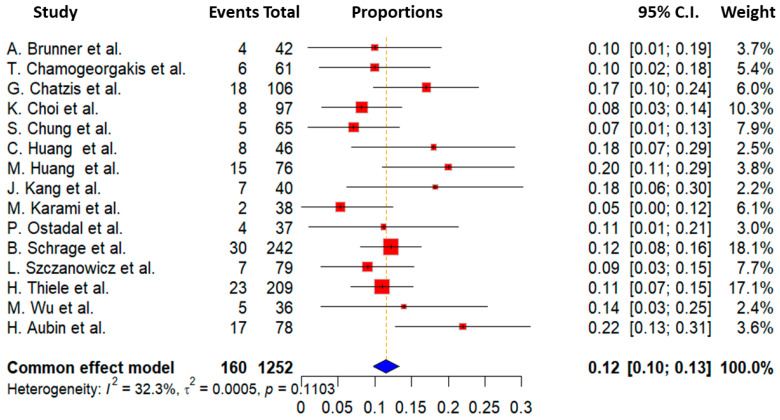
Forest plot of studies included in the outcome of ischemia complication [7,11,13,15,16,18,19,21,22,25,27,29,30,31,32].

**Figure 4:**
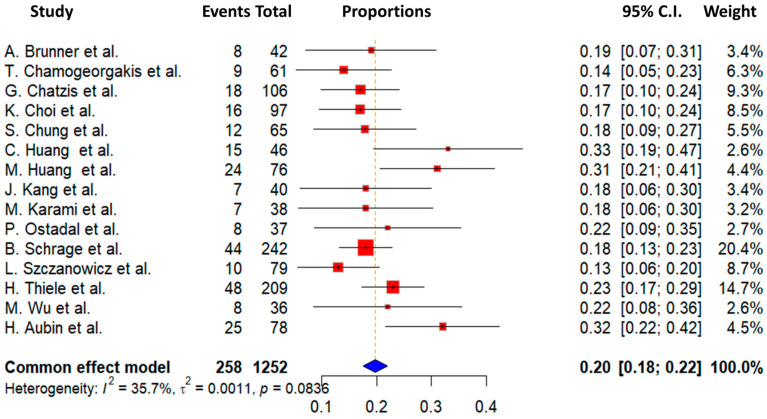
Forest plot of studies included in the outcome of bleeding complication [7,11,13,15,16,18,19,21,22,25,27,29,30,31,32].

In this study, we also focused on examining the occurrence of bleeding and ischemia associated with the use of ECMO devices as secondary endpoints. Regrettably, data from all studies were not available, but we were able to obtain and analyze these outcomes in the majority of the studies included. Information on ischemia and bleeding events was accessible in 15 out of the 24 studies included in the meta-analysis, involving a total of 1295 patients out of 4706 participants (27.5% of the total cohort). The overall ischemia rate averaged 12.9 ± 5.1%, with a range from 7.1% to 22%. The lowest ischemia incidence was documented in the study by Chung et al. at 7.1%, while the highest was observed in the research conducted by Aubin et al., with an incidence of 22%. Regarding bleeding complications, we found an overall incidence of 21 ± 6.9%. Bleeding rates varied from 3.2% to 33%, with the lowest reported by Chamogeorgakis et al. [11] and the highest by C. Huang et al. [18] Detailed information on ischemia and bleeding events are demonstrated in Figure 3 and Figure 4, respectively.

## 4. Discussion

The primary findings of our meta-analysis are summarized as follows:

(a) The 30-day mortality rate for cardiogenic shock patients is still high even with the use of VA-ECMO;
(b) Our findings may, however, imply that vascular complications are linked to a worse prognosis.

Since the implementation of PCI, there have been no significant improvements in CS outcomes caused by ACS attributable to specific materials such as the usage of specific drugs, procedures, or related conventions, although PCI has certainly improved the short-term outcomes of treated patients [33]. As mortality rates continued to be significant, additional technologies were introduced to enhance outcomes. Multiple randomized trials did not demonstrate any prognostic advantage of pressure-reducing devices for the left ventricle, such as an intra-aortic balloon pump [34], while devices like Impella, which reduces the volume of the left ventricle, showed promise in improving the prognosis [35]. ECMO, another form of MCS, has also proven to be effective in treating patients with refractory CS [36,37].

Based on our findings, the 30-day mortality rate among patients with CS who received VA-ECMO treatment aligns with existing research [30]. These data indicate that improving prognosis through increased cardiac output, resulting in a reduced risk of multiple organ failure and progression to cardio-metabolic shock, may be achieved using this device. However, when comparing our study’s mortality rate to other available data, no significant decrease was observed. Several potential explanations for this discrepancy are discussed below.

As mentioned previously, the participants in this meta-analysis were specifically individuals who suffered from CS related to ACS. This condition has been linked to a higher rate of mortality compared to cases of acute myocardial infarction without CS [38]. Various intricate factors need to be considered when examining this group of patients, such as the timing of CS in relation to ECMO implantation, the initiation of coronary intervention and its outcomes in terms of TIMI-Flow achievement, the occurrence of cardiopulmonary resuscitation, and the time to return of spontaneous circulation (ROSC) in certain patients. The expertise of the interventionist, the quality of the procedure, and the speed of achieving results are also crucial aspects to account for. Additionally, the management of metabolic acidosis, pulmonary failure, biventricular insufficiency, and the potential need for escalated support through other MCS like Impella are significant factors influencing the patients’ outcomes during subsequent critical care procedures in the intensive care unit. It is also worth noting that VA-ECMO therapy can lead to increased afterload on the left ventricle, potentially worsening ventricular dysfunction related to the heart attack [39]. Given the potential impact of multiple variables, including factors like inflammation and coronary and cerebral oxygen deprivation on mortality rates, it is essential to acknowledge the complex interplay among these elements rather than attributing all outcomes solely to the efficacy of ECMO treatment.

Discussion of certain biases is essential in this context. Firstly, it is important to address the impact of confounding variables, as the combined influence of the mentioned factors could potentially affect the mortality rate target differently than each factor individually, potentially exhibiting synergism with an additive or multiplicative impact. Secondly, potential effect modification by variables like gender and age should be taken into account. These factors may follow unique biological mechanisms that cannot be accurately captured by either multiplicative or additive models. Thirdly, the possibility of publication bias needs to be acknowledged. Relevant studies suitable for inclusion in this meta-analysis may exist from a qualitative standpoint that remain unpublished, are awaiting publication, or are only available in obscure journals, thereby being unknown to the analysis. Although evidence provided from funnel plots is not definitive, the funnel plot analysis for all outcomes measured, such as mortality, ischemia, and bleeding, shows no publication bias in our meta-analysis (Supplementary Materials, Figures S1–S3). Furthermore, this meta-analysis encompassed multicenter studies, but the extent of variations in inclusion/exclusion criteria, treatment regimens, and diagnostic protocols across these studies remains unclear. Moreover, it is crucial to emphasize that the evaluation in this research is grounded on meta-regression, serving as the fundamental element of the results, as opposed to direct contrasts between treatments.

Another crucial aspect to be addressed is the pronounced heterogeneity observed in the forest plot depicting mortality rates (Figure 2). Several factors may account for this variance. Notably, lactate levels were inconsistently documented across the studies, with values spanning a wide range of 5.6 to 14 mmol/L, indicating patients at varying stages of CS undergoing VA-ECMO treatment. Lactate levels are recognized as independent predictors of early mortality in such patients [40]. Similarly, the range of left ventricular ejection fractions (21% to 45%) at the time of VA-ECMO intervention varied significantly, serving as another independent predictor of early mortality rates [41]. Furthermore, the substantial variability (24% to 81%) in the incidence of cardiopulmonary resuscitation among the different patient cohorts underscores the diverse clinical conditions experienced by the patients. The duration of resuscitation was inconsistently reported across studies, further highlighting the varying circumstances in which patients were treated.

This meta-analysis provides added insights into the realm of complications stemming from device use. The incidence rates of major vascular ischemic complications and major bleeding were recorded at 12.9% and 21%, respectively. These results are consistent with earlier research findings [30]. Plausible reasons for these outcomes encompass various factors: the accurate evaluation of dedicated access is compromised when contrasting elective procedures against urgent settings, typically encountered in scenarios of CS. The critical aspect of selecting the appropriate sheath-to-femoral artery ratio becomes evident due to the utilization of large French sizes with VA-ECMO. Operator proficiency in puncture technique and effective management of broader access pathways also significantly influence the outcomes. The observed high mobility rate in ACS with CS could potentially be attributed to the inclusion of vascular complications solely in the analysis, since there was a direct linear correlation between mortality rates and major ischemia and bleeding incidence, at least across studies reporting these complication rates. The elevated complication rate noted in multicenter studies with larger participant pools could be attributable to the involvement of sites with limited device experience, potentially skewing the overall results.

### Limitations

This is not a patient-generated data analysis; rather, the data utilized came from observational retrospective studies, so the results are to be considered just as hypothesis and not as solid evidence of causality. Furthermore, we utilized a common effect model to estimate total mortality across the studies. While a random effect model might have been more suitable for this analysis, we opted not to use it, as our primary objective was to provide a descriptive overview of the literature on the specific topic of acute coronary syndrome (ACS)-related cardiogenic shock (CS) managed with VA-ECMO. This decision was made despite the potential for bias, prioritizing clarity and simplicity in presenting the data.

A special limitation of the current analysis is the fact that there was no comparison of ECMO with patients without mechanical support in the context of infarct-associated CS, so that the net therapeutic effect of VA-ECMO on mortality in this setting cannot be clearly determined. The objective of the present meta-analysis was to examine, in a descriptive manner, the impact of VA-ECMO on mortality and complications in the context of infarct-related CS. We did not intend to investigate the therapeutic effect of VA-ECMO as a treatment for CS. While such an analysis is indeed of significant interest, the available data on patients treated without mechanical support in the setting of infarct-related CS are limited and outdated, thus preventing a fair comparison between the groups.

The precise time latency between the onset of CS and VA-ECMO implantation as well as the precise timing of the coronary interventions could not be found in all the studies included in this analysis, so the significant differences between the studies may result from different inter-clinical procedure algorithms. To further assess the influence of the lactate level, ejection fraction, or the inotropes that may have a major impact on the results, meta-regressions involving sufficient data were not available from the included studies.

## 5. Conclusions

The use of VA-ECMO has become a life-saving intervention for patients with refractory CS. However, our findings reveal a high mortality rate of 63%, reflecting both the critical condition of these patients and the limitations of the therapy. This highlights the need for early identification of suitable candidates, meticulous patient selection, and optimized peri-ECMO management to improve outcomes. Additionally, the results underscore the importance of developing standardized protocols, improving complication-prevention strategies and advancing VA-ECMO technology to enhance survival. Despite its life-saving potential, the high mortality rate associated with VA-ECMO calls for ongoing efforts to refine its application and maximize therapeutic benefit.

### Future Directions

The mortality of CS complicated by an ACS in patients treated with a VA-ECMO remains unacceptably high, mainly driven by the high ischemia and bleeding complication rates. Possible aspects for improving the outcome in patients with ACS complicated by CS could be the selection of the right device in the right patient with timely implantation and implementation of ubiquitous procedural steps in the form of shock team networks in dedicated centers to firstly ensure maximum safety for the patient and secondly to achieve better comparability of the acquired data for future studies to better understand the research of this clinical setting.

## Figures and Tables

**Table 1:** Presentation of included studies in the present meta-analysis.

First Author	Publication Date	Journal	Study-Design	Clinical Endpoint
Aso [10]	2016	Crit Care Medicine	Retrospective Multicenter	30-Day mortality
Brunner [7]	2019	JACC	Prospective Singlecenter	30-Day mortality
Chamogeorgakis [11]	2013	ASAIO Journal	Retrospective	In-Hospital outcome and long-term survival
Chang [12]	2016	Circulation	Retrospective	30-Day and one year survival
Chatzis [13]	2021	Clinical Research in Cardiology	Retrospective	In-Hospital and 6-months survival
Cho [14]	2018	KCJ	Retrospective	30-Day mortality
Choi [15]	2020	Circulation Journal	Retrospective	In-Hospital mortality and 1-year follow-up
Chung [16]	2013	Int. J. Cardiol.	Retrospective	30-Day mortality
Fried [17]	2021	ASAIO Journal	Prospective Singlecenter	Survival to discharge and 30-Day survival
C. C. Huang [18]	2018	Int. J. Cardiol.	Retrospective	Six-month survival
M. Huang [19]	2020	ASAIO Journal	Retrospective	30-Day mortality
Hyun [20]	2023	Cardiology Journal	Prospective Multicenter	3-Years monitoring and follow-up
Kang [21]	2024	ESC Heart Failure	Retrospective	90-Day mortality
Karami [22]	2020	European Heart Journal Acute Cardiovascular Care	Retrospective	30-Day mortality
Kim [23]	2021	KCJ	Retrospective Multicenter	30-Day mortality
Mousa [24]	2022	Critical Care	Retrospective	30-Day mortality
Ostadal [25]	2023	Circulation	Retrospective Multicenter	30-Day mortality
Pozzi [26]	2023	Int. J. Cardiol.	Retrospective Multicenter	90-Day Mortality
Schrage [27]	2020	Circulation	Retrospective	30-Day mortality
Shin [28]	2021	Medicina	Retrospective	30-Day mortality
Szczanowicz [29]	2021	JIC	Prospective Multicenter	6-months survival
Thiele [30]	2023	NEJM	Prospective Multicenter	30-Day mortality
Wu [31]	2014	Resuscitation	Retrospective	Survival to discharge and LT-follow-up
Aubin [32]	2016	JACC	Retrospective	Survival to discharge and 2-year-follow-up

JACC: Journal of American College of Cardiology; ASAIO: American Society Organ for Artificial Internal Organs; ESC: European Society of Cardiology; KCJ: Korean Circulation Journal; JIC: Journal of Invasive Cardiology; NEJM: The New England Journal of Medicine; JACC: Journal of the American College of Cardiology.

**Table 2:** Analysis of the included studies in the meta-analysis with their characteristics.

First Author	Number of Participants	30-Day Mortality (%)	Age (y)	Male (%)	Bleeding (%)	Lactate (mmol/L)	LVEF (%)	Prior CPR (%)	pH	Creatinine (mg/dL)
Aso [10] *	473	62	n.a.	69.4	n.a.	n.a.	n.a.	n.a.	n.a.	n.a.
Brunner [7]	42	33	62 (50–68)	76	19	n.a.	n.a.	n.a.	n.a.	n.a.
Chamogeorgakis [11]	61	50	53 ± 13	49	14	n.a	n.a.	33%	n.a.	n.a.
Chang [12] *	1705	66	57 ± 17	70.6	n.a.	n.a	n.a	n.a	n.a	n.a
Chatzis [13]	106	56	61.3 ± 10.2	78	17	9.18 ± 5.6	35 ± 4.5	n.a.	7.2 ± 0.2	1.47 (1.3–1.9)
Cho [14]	42	78.6	63 ± 11.4	65	n.a.	7.4 ± 5	27 ± 13.6	n.a.	7.2 ± 0.2	1.35 ± 1.1
Choi [15]	97	48	63.8 ± 12	78	17	5.2 ± 4.4	n.a.	60%	n.a.	1.7 ± 1.3
Chung [16]	65	43	60.1 ± 7.6	89	17.9	n.a.	n.a.	n.a.	n.a.	1.77 ± 1.62
Fried [17]	109	56	60 ± 11.7	73.8	n.a.	5.95 ± 4.84	21	n.a.	7.28 ± 0.16	1.64 ± 0.87
C. C. Huang [18]	46	63	57 ± 11.2	87	33	n.a.	n.a.	n.a.	n.a.	n.a.
M. Huang [19] *	76	34	45 (34–58)	79	31	n.a.	n.a.	n.a.	n.a.	n.a.
Hyun [20]	104	74	63.3 ± 11.8	77.9	n.a.	n.a.	34 ± 14	n.a.	n.a.	1.6 ± 1.9
Kang [21] *	40	50	68 (59–75)	64	18	8	30 (20–40)	46%	n.a.	1.3 (1–2)
Karami [22]	38	53	55 ± 9	79	18	7.1 ± 4.8	n.a.	24%	7.2 ± 0.2	1.33 (1–1.8)
Kim [23]	184	80.4	60.5 ± 12.2	88	n.a	n.a.	n.a.	47%	n.a.	n.a.
Mousa [24] *	121	48	55.9 ± 13.8	67.8	n.a.	n.a.	n.a.	n.a.	n.a.	n.a.
Ostadal [25] *	37	50	67 (60–74)	74.1	22	5.3 (3.1–8.4)	n.a.	n.a.	n.a.	n.a.
Pozzi [26] *	649	61	57.1 ± 10.4	80	n.a.	n.a.	n.a.	n.a.	n.a.	n.a.
Schrage [27] *	242	62	57.5 ± 13	77.7	18	8.9	n.a.	66%	7.18 ± 0.2	n.a.
Shin [28] *	67	72	65 (55–77)	76.1	n.a.	n.a.	24 (16–39.5)	66	n.a.	1.1 (1–2)
Szczanowicz [29] *	79	62	60 ± 11	n.a.	13	8.2	n.a.	n.a.	7.2	1.6
Thiele [30]	208	47.8	62 (56–69)	81.3	23	n.a.	30 (20–35)	78%	7.2(7.1–7.3)	1.2 (1–1.5)
Wu [31] *	36	92	68 (40–83)	78	22	n.a.	45 (28–73)	81%	n.a.	n.a.
Aubin [32] *	78	58	56 ± 15.2	75	32	n.a.	n.a.	79%	n.a.	n.a.

y: years of age; LVEF: left ventricular ejection fraction, CPR: cardiopulmonary resuscitation, n.a.: not available. Data are presented as mean ± standard deviation or median (25th–75th percentile) or as percentages (%). * Data presented are listed after special request from corresponding authors or after adjustment according to values given in the studies. Minimal deviations cannot be excluded.

## Data Availability

The data that support the findings of this study are available from the corresponding author upon reasonable request.

## Supplementary Materials

The following supporting information can be downloaded at: https://www.mdpi.com/article/10.3390/biomedicines13010237/s1, Figure S1. Funnel plot of studies of the meta-analysis on mortality outcome; Figure S2. Funnel plot of studies of the meta-analysis on ischemia outcome; Figure S3. Funnel plot of studies of the meta-analysis on bleeding outcome.
