# Supplementary figures and images for: Venoarterial Membrane Oxygenation in Cardiogenic Shock Complicated from an Acute Myocardial Infarction: An Overview and Comprehensive Meta-Analysis
Source: Biomedicines. 2025 Jan 20;13(1):237. doi: 10.3390/biomedicines13010237 (PMC11760826; doi:10.3390/biomedicines13010237)

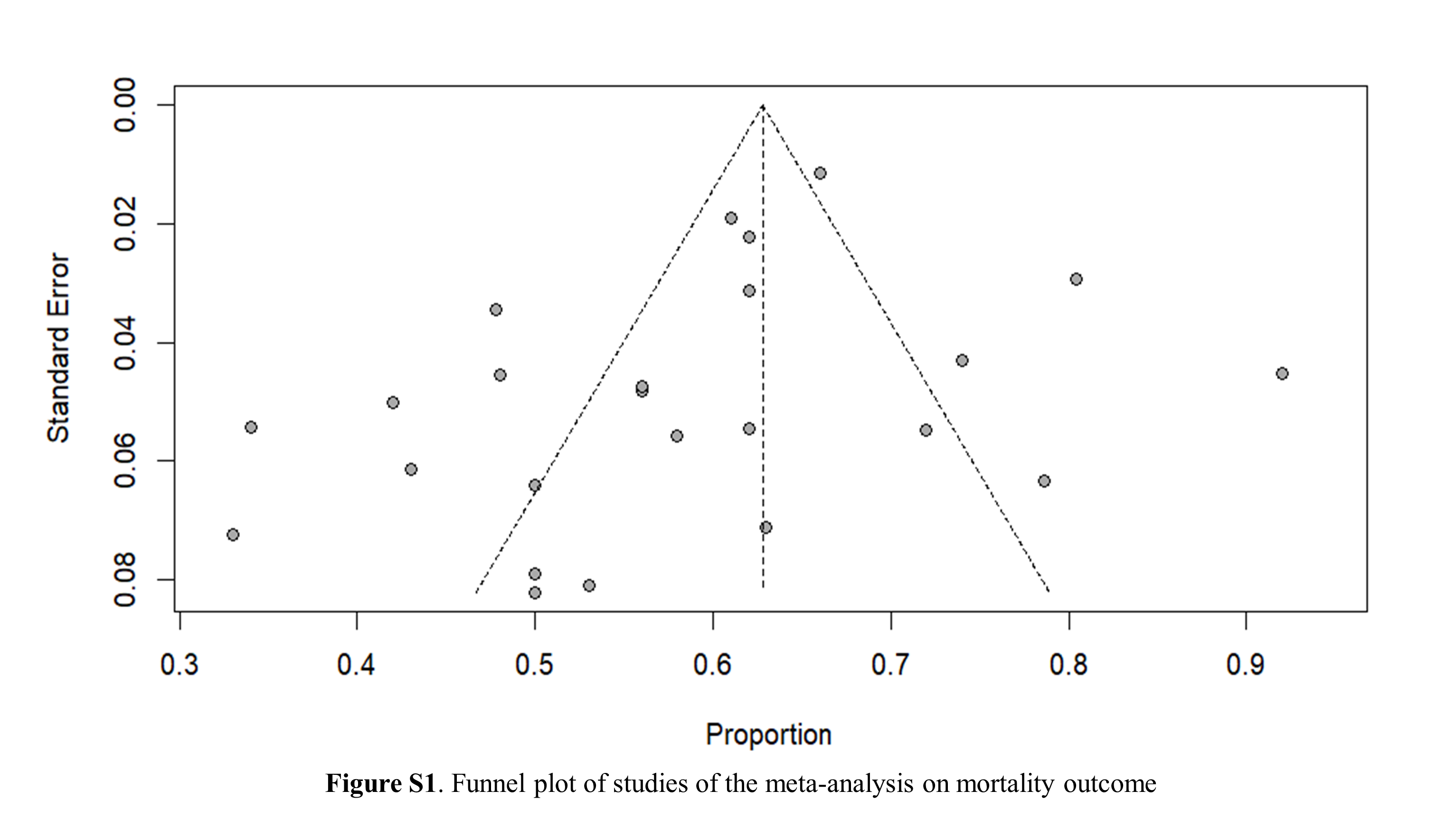

Supplement: Supplementary file 1 [file biomedicines-13-00237-s001.zip › Figure S1.tif]

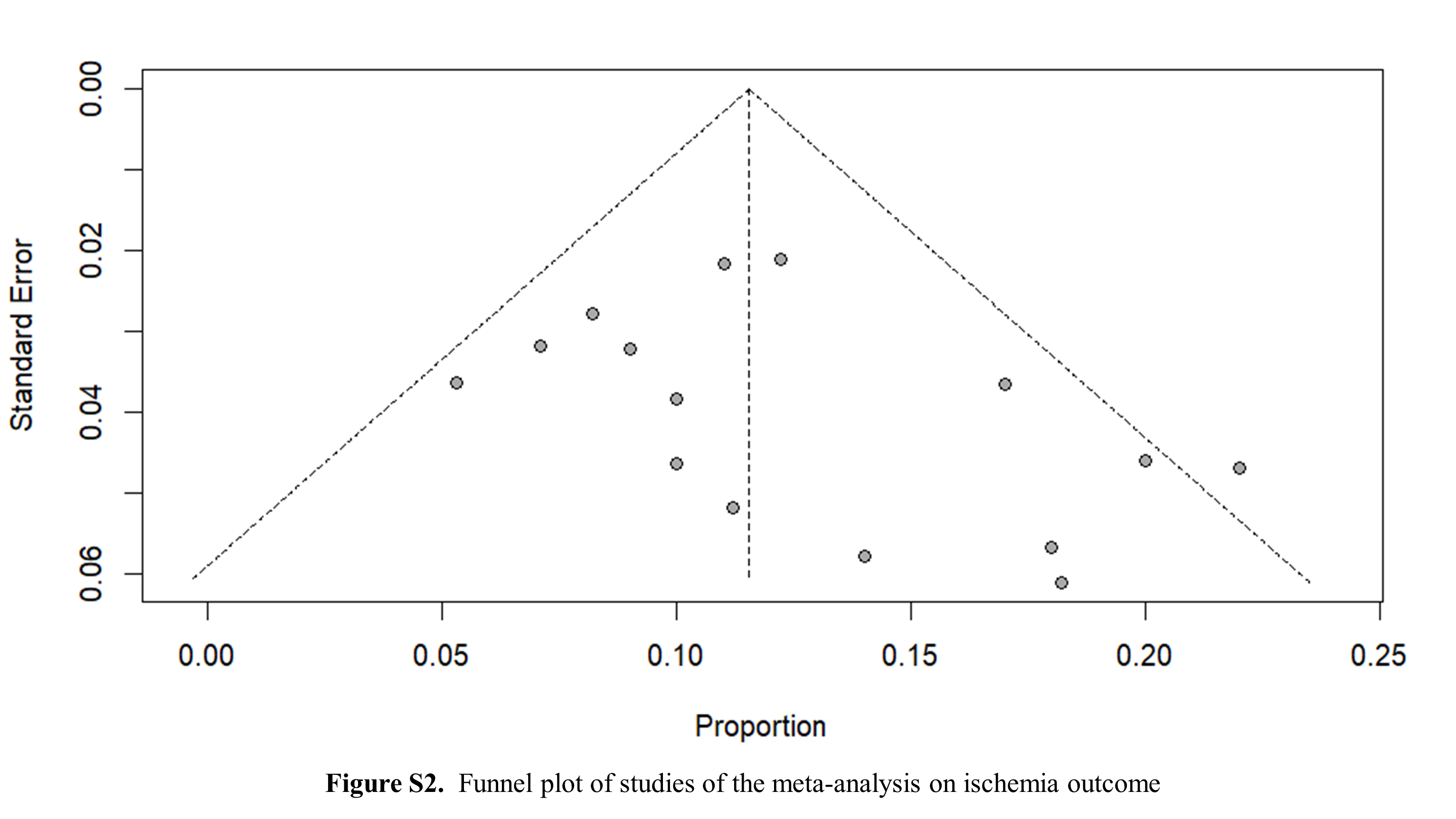

Supplement: Supplementary file 1 [file biomedicines-13-00237-s001.zip › Figure S2.tif]

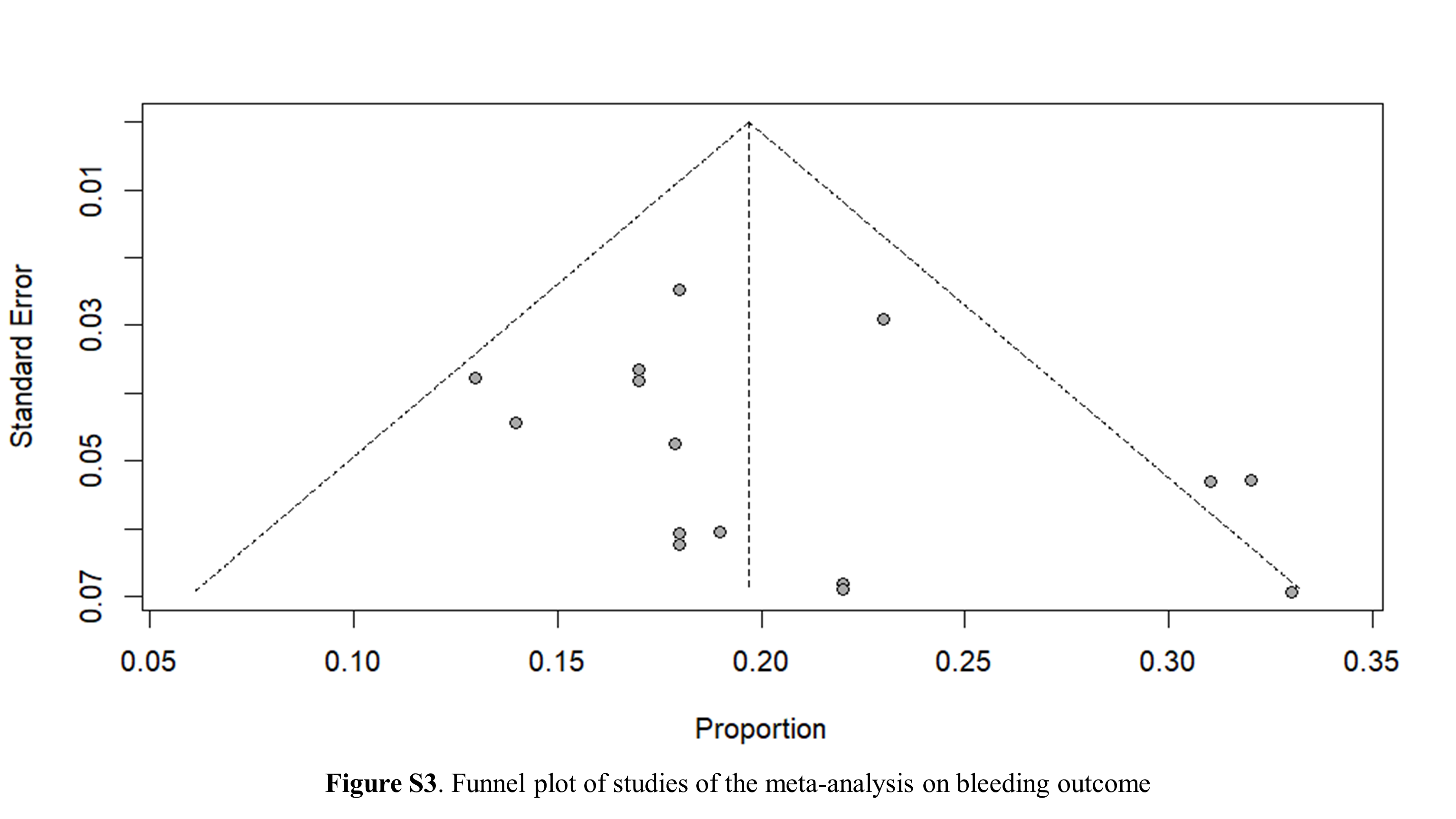

Supplement: Supplementary file 1 [file biomedicines-13-00237-s001.zip › Figure S3.tif]
